# Military veterans with mental health problems: a protocol for a systematic review to identify whether they have an additional risk of contact with criminal justice systems compared with other veterans groups

**DOI:** 10.1186/2046-4053-1-53

**Published:** 2012-11-06

**Authors:** James Taylor, Tessa Parkes, Sally Haw, Ruth Jepson

**Affiliations:** 1 School of Nursing Midwifery and Health, University of Stirling, Stirling, Scotland, UK

**Keywords:** Military, Veterans, PTSD, Criminal justice, Substance misuse, Mental health, Offender, Alcohol use

## Abstract

**Background:**

There is concern that some veterans of armed forces, in particular those with mental health, drug or alcohol problems, experience difficulty returning to a civilian way of life and may subsequently come into contact with criminal justice services and imprisonment. The aim of this review is to examine whether military veterans with mental health problems, including substance use, have an additional risk of contact with criminal justice systems when compared with veterans who do not have such problems. The review will also seek to identify veterans’ views and experiences on their contact with criminal justice services, what contributed to or influenced their contact and whether there are any differences, including international and temporal, in incidence, contact type, veteran type, their presenting health needs and reported experiences.

**Methods/design:**

In this review we will adopt a methodological model similar to that previously used by other researchers when reviewing intervention studies. The model, which we will use as a framework for conducting a review of observational and qualitative studies, consists of two parallel synthesis stages within the review process; one for quantitative research and the other for qualitative research. The third stage involves a cross study synthesis, enabling a deeper understanding of the results of the quantitative synthesis. A range of electronic databases, including MEDLINE, PsychINFO, CINAHL, will be systematically searched, from 1939 to present day, using a broad range of search terms that cover four key concepts: mental health, military veterans, substance misuse, and criminal justice. Studies will be screened against topic specific inclusion/exclusion criteria and then against a smaller subset of design specific inclusion/exclusion criteria. Data will be extracted for those studies that meet the inclusion criteria, and all eligible studies will be critically appraised. Included studies, both quantitative and qualitative, will then undergo stage-specific analysis and synthesis. The final stage will combine the findings of both syntheses to enable new understandings of why, how, and by how much, military veterans with mental health problems, including problematic drug and alcohol use, come into contact with the criminal justice system.

## Background

The military conflict in Afghanistan has, once again, brought to the attention of politicians, the public and the press, the dangers of serving in the armed forces and the difficulties the personnel experience once they have finished active service. There is concern that a proportion of armed forces personnel experience problems when leaving military service and returning to civilian status [1]. While the majority of armed forces personnel manage this transition [2], some experience a range of difficulties. These include mistrust [3], unemployment [4], particularly for those over 25 years of age [5,6], boredom, a lack of money [7], homelessness [8,9], on-going poor mental health [2,6,10,11], and suicidality [12-14]. While the definition of ‘military veteran’ differs between countries (see Dandeker *et al.* for an overview of definitions [15]), concern has also been voiced that the complex needs of recently deployed military veterans in both the United States (US) [16] and the United Kingdom (UK) [7] could increase their future contact with criminal justice services.

Since the mid-1980s, when the number of veteran prisoners peaked at 21% of all US prisoners, there has been a slow decline in the number of incarcerated military veterans [17]. The last available figures report that 10% of US Federal and State prisoners are veterans [18], however this relates to data gathered in 2004. Within the UK, and while the exact number of veterans is probably unknown [19], the Home Office suggested that at the beginning of the last decade around 3% of prisoners were military veterans [20]. More recent figures for the number of veterans in prison range from 3.5% [21] to 8.5% [22]. Figures proposed for the number of people supervised by UK probation services who have veteran status also differ ranging from 3.4% [23] to 6% [24]. Such disparity indicates a degree of uncertainty over the actual number of veterans in UK prisons.

Although societal challenges, such as soldiers returning from war, and different periods in time may influence the risk of veteran imprisonment [25], debates on whether military service causes future offending have been longstanding. For example, accounts of such arguments are evident after the Second World War [26]. However, more recently, Bouffard [27] found no relationship between military service and subsequent criminal or violent behavior, finding instead that military service reduced future criminality. Conversely, Galiani and colleagues [28] found that conscripted military service is positively related to future criminal behavior and conviction. These contrary views may be attributable to the ‘type’ of person engaged in military service and not the service *per se*. Personal characteristics and the ‘quality’ of the individual, such as educational attainment, anti-social traits or mental health problems [29], may have stronger influences on the likelihood of future offending and incarceration than the engagement of military service [27,30].

Military veterans experiencing mental health difficulties, particularly post-traumatic stress disorder (PTSD) and combat related stress, is not a new phenomenon [31,32]; however, veterans with mental health difficulties can find themselves imprisoned [29]. Black and colleagues [29] found that incarcerated veterans had a higher frequency of psychiatric illness than non-imprisoned veterans. They also found that veteran incarceration was associated with high healthcare utilization and contact with mental health professionals.

Military veterans with PTSD [33-35] or combat experience [29] may find themselves incarcerated, yet the associations between PTSD or combat exposure and imprisonment are not without ambiguity [30,33,36]. Despite the uncertainty of a direct relationship with imprisonment, combat exposure has been strongly associated with aggressive tendencies [33], drug use [37], alcohol consumption [38], and engaging in risk taking behaviors [39]. Further, while veterans with PTSD may find themselves imprisoned, the prevalence of PTSD in military personnel and veterans shows variability across countries [40]. For UK veterans PTSD is less common than depression [4], and both depression and alcohol abuse appear more problematic [41].

Alcohol use is part of the social fabric of some armed forces and alcohol problems within the military are not a new phenomenon. Wagley [42] comments with concern on the number of military offenders with alcohol problems requiring offender rehabilitation post World War II. More than half a century later excessive alcohol use by military personnel is still evident. Excessive use of alcohol has been found to be more common in UK military personnel than the UK general population [43], with alcohol misuse the most common mental health problem found in new military veterans [41]. Alcohol and other drugs may be used to gain relief from, and cope with, the psychological consequences of combat exposure [3,34]. For example, a relationship exists between excessive alcohol use and combat exposure [38,44,45]. Alcohol misuse in veterans can also contribute to imprisonment [41,46].

While alcohol misuse is common across veteran age groups, some younger veterans are also using drugs [47]. Substance use was evident among military personnel during the Vietnam War [34] and, while the majority stopped such use following discharge [48], small numbers continued [33,49]. It is recognized that substance use can contribute to the incarceration of veterans [17,18,46,50]. For example, incarcerated Vietnam veterans were more likely to have substance use problems than their non-convicted counterparts [33], use which might not be attributable to their military service. Veterans who continued opiate use after returning from Vietnam (and after ending their military career) tended to, among other factors, have pre-enlistment substance use and engagement in deviant activities [49].

In summary, despite current concerns, veteran contact with criminal justice systems is not a new phenomenon and a number of contributory factors have been reported. Previous research, as discussed above, has identified veteran poor mental health, alcohol and substance use, and the consequences of exposure to combat as having an impact on veterans returning to a civilian life. These may also contribute to their contact with criminal justice systems. However, there is no consensus on this and other reasons have been suggested. This review looks to resolve this by identifying whether the above are contributory factors to military veterans having contact with criminal justice systems and whether such factors provide an additional risk to said contact when compared with mentally healthy military veterans.

### Aims and objectives

The primary objectives of the review are to:

• Synthesize the evidence on the amount and type of contact with criminal justice systems for those military veterans with mental health problems, including substance use compared to those veterans who do not have such problems.

• Synthesize the evidence on the views and experiences of military veterans with mental health and/or substance misuse problems on their experiences regarding contact with criminal justice services and what they perceived contributed to, or influenced, their contact with said services.

• Use the synthesis of the qualitative studies to illuminate and explain the results from the quantitative synthesis.

In addition to the above, and with specific reference to military veterans with mental health problems, the review will also seek to address the following supplementary questions:

• are there differences in types of criminal justice contact or military experience and;

• are there international and temporal differences in veteran contact with criminal justice services, and if so how do these contact types differ.

If studies are available international comparisons will focus on the US, UK, Australia, New Zealand, Canada and European Union Countries. Comparison of temporal differences will focus on the immediate years after key conflict periods, namely World War 2, Korean War, Vietnam War, Falklands Conflict, 1^st^ Gulf War, 2^nd^ Gulf War and the Afghanistan conflict.

## Methods/design

**Figure 1:**
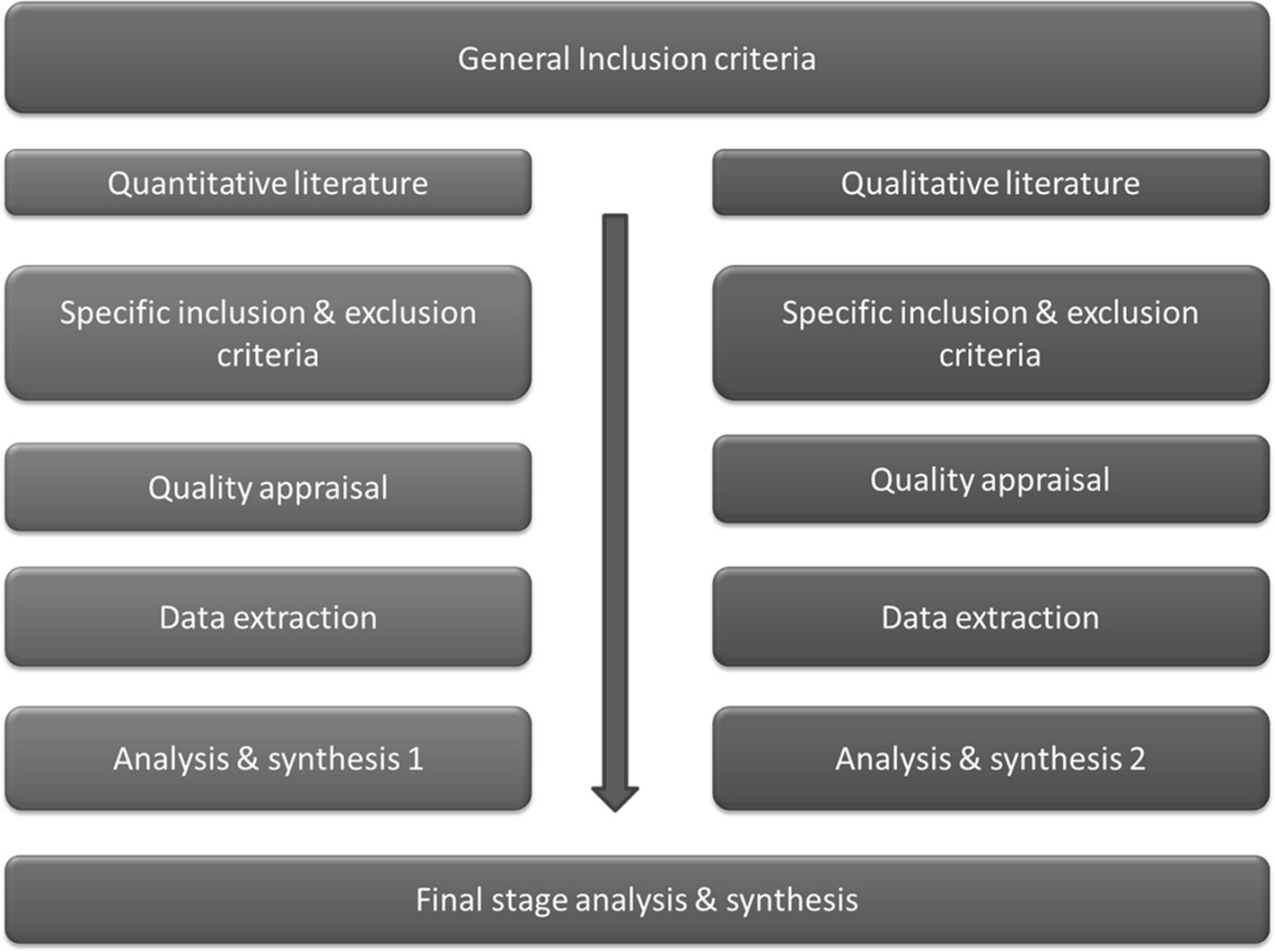
Methodological process for systematic review.

While the practice of conducting a systematic review traditionally involves a discrete linear process [51], this review will adopt a process similar to the model proposed by Harden and Thomas [52]. While Harden and Thomas’ model was used for conducting systematic reviews of intervention studies, their model will be extended in this current study and used as a framework for conducting a review of observational studies. Harden and Thomas’ approach consists of two parallel sets of stages in the review process: one for quantitative research and the other for qualitative research. The parallel stages each contain distinct inclusion criteria, data extraction processes and quality assessment. This review emulates much of the model proposed by them, however one significant change adopted is that the parallel stages will be screened against a general set of inclusion/exclusion criteria and then against a smaller subset of stage specific inclusion criteria. The parallel stages will undergo individual analysis and synthesis and, where possible, include a final synthesis stage combining the findings of the previous analysis and synthesis, as proposed by Harden and Thomas. Figure 1 provides a diagrammatical overview of this study’s methodological model.

### Criteria for selecting articles/studies for this review

The systematic review will consist of automated and manual search strategies. The initial selection criteria will be broad to ensure as many studies as possible are identified for initial screening. General, topic specific, inclusion and exclusion criteria, as defined below, will then be applied to titles and abstracts for the purpose of screening. This will be conducted independently by two members of the project team. Full articles and reports will be obtained for those documents that meet the general inclusion criteria or where there is insufficient information available to exclude the document at screening. Full articles and reports will then be reviewed against the general inclusion/exclusion criteria and then against the stage/design specific inclusion criteria, independently by both team members. Where differences of opinion occur regarding inclusion eligibility resolution will be sought through discussion.

### General inclusion criteria

• All articles and reports must include military veterans who are no longer in active service or who are reservist or territorial personnel who have experienced deployment but have now returned to civilian life;

• The UK definition of veteran will be adopted irrespective of paper geographical location or sample nationality, that is, must have served one day in an armed force;

• Military veterans with ‘honorable’, ‘dishonorable’ and ‘medical discharges’ will be included;

• Veterans must have mental health problems. Mental health problems will include those with substance use problems;

• Mental health problems will be defined as those that would meet, on appraisal, categorization in the following International Classification of Diseases, 10th revision (ICD-10) classifications [53]:

F20-29 Schizophrenia, schizotypal and delusional disorders

F30-39 Mood (affective) disorders

F40-48 Neurotic, stress related and somatoform disorders

• Where an article predates the publication of ICD-10 then the authors will match the clinical presentation described with one of the modern classifications. For example, war neurosis, combat fatigue, shell shock, hysteria, psychoneurosis and anxiety reaction would be matched with the ICD-10 F40-48 classification; and manic depression and reactive depression would be matched with the ICD-10 F30-39 classification.

• Substance use problems will include alcohol problems and dependence, and other psychological and behavioral problems associated with alcohol use;

• Substance use will include regular illicit drug use, drug dependence and misuse of prescription medication, as well as other psychological and behavioral problems associated with substance use;

• Criminal justice systems will include court services, probation services, correctional, young offender and prison services, and other remand or post-sentence custodial or secure environments, for example, secure mental health facilities.

### General exclusion criteria

• Reports and articles that focus only on police arrests and police cautions;

• Reports and articles that only address military veterans, reservist and territorial personnel with diagnosed anti-social personality disorders;

Reports pre-dating the onset of World War 2, that is, prior to 1939;

• Articles or reports that are wholly descriptive, where there is no evidence of either qualitative or quantitative structured inquiry;

Material not in English;

• Articles or reports that primarily focus on the physical health consequences of alcohol or substance use.

### Quantitative stage specific inclusion criteria

• Studies reporting on the prevalence and/or incidence of veterans with mental health and/or substance use problems;

• Studies reporting on the prevalence and/or incidence of reservists or territorial army personnel with mental health problems and/or substance use problems;

• Studies reporting on the prevalence and/or incidence of veterans or reservists/territorial army personnel engaged with criminal justice systems, as defined in the general inclusion criteria;

• Empirical case–control and cohort studies comparing military veterans, including reservists, with and without mental health problems, who have and have not had contact with criminal justice systems.

### Quantitative stage specific exclusion criteria

Studies detailing mental health or substance use data obtained prior to joining military service where no empirical case–control or cohort post service comparison is available;

Case–control or cohort studies obtaining mental health or substance misuse data from reservists/territorial army personnel during screening for deployment where no previous deployment has occurred;

Studies that only focus on the testing of psychometric properties of measuring tools for detecting mental health problems;

Studies where mental health problems have not been clinically confirmed.

### Qualitative stage specific inclusion criteria

• Focus group or interview studies reporting on the views, opinions and experiences of military veterans with mental health problems, irrespective of model of qualitative analysis used;

• Focus group or interview studies reporting on the views, opinions and experiences of reservist or territorial army personnel with mental health problems, irrespective of model of qualitative analysis used.

### Qualitative stage specific exclusion criteria

• Single case studies;

• Studies examining the opinion and views of territorial or reservist personnel pre-deployment where no previous deployment or post deployment analysis has occurred;

• Studies reporting only on the views, opinions and experiences of criminal justice worker contact with military veterans;

• Studies examining qualitative methodological issues only.

### Search strategy for identification of articles

Sets of database search terms/keywords will cover the four concepts: criminal justice, military veterans, mental health, and substance use. International reports and articles will be included in the review; however, all papers must be written in English or have a published English language translation. All databases will be searched up to the end of November 2011 from either the date of commencement of the database archive or 1939. Databases to be used for automated searching are: Web of Science, Medline, CINAHL, Health Source Nursing Academic Edition, Psych Info, Psych Articles, National Criminal Justice Reference Service Abstracts, PILOTS Database Abstracts, Social Services Abstracts, Sociological Abstracts and The Cochrane Database of Systematic Reviews. Table 1 describes the search structure and lists the keywords used during the literature search. Table 2 lists the Medical Subject Headings (MeSH) that will be searched. In addition to searching the formal databases defined above, combined keyword searches will be conducted in Google Scholar and Google Web and manual searches will be conducted on the following websites:

• DASA

• The Royal British Legion

• The Howard League for Penal Reform

• Scottish Prison Service

Scottish Government

• UK Ministry of Justice and National Offender Management Service

• United States Department of Veteran Affairs Justice

• United States Department of Justice

• Australia Government Department of Veteran Affairs

• Social Sciences Research Network

• Prison Health Research Network

• School of Forensic Mental Health

**Table 1:** Example of database search terms and Inquiry structure

**Database search terms/keywords and search structure**				
1) substance misuse	2) drug use	3) illicit medic*	4) narcotic*	5) medication abuse
6) #1 OR #2 OR #3 OR #4 OR#5	7) alcohol	8) alcoholic beverages	9) inebriant	10) intoxicant
11) #7 OR #8 OR #9 OR #10	12) mental health	13) mental illness	14) psychiatr*	15) depress*
16) PTSD	17) Traum*	18) #12 OR #13 OR #14 OR #15 OR #16 OR #17	19) #6 OR #11 OR #18	20) veteran*
21) ex-military	22) $military	23) armed force*	24) soldier	25) army
26) navy	27) marine	28) air force	29) military	30) #20 OR #21 OR #22 OR #23 OR #24 OR #25 OR #26 OR #27 OR #28 OR #29
31) prison*	32) incarcerat*	33) custody	34) jail	35) gaol
36) offender	37) criminal	38) inmate*	39) probation*	40) law enforce*
41) legal	42) court	43) justice	44) police	45) sentence
46) correction*	47) #31 OR #32 OR #33 OR #34 OR #35 OR #36 OR #37 OR #38 OR #39 OR #40 OR #41 OR #42 OR #43 OR #44 OR #45 OR #46	48) #30 AND #47	49) #19 AND #48	

**Table 2:** MeSH headings to be used during search

**MeSH headings**		
Veterans	Veterans Health	Military Personnel
Military Psychiatry	Military Medicine	Prisoners (and subheadings)
Prison	Drug users	Substance related disorders
Street Drugs	Alcohol related disorders	Alcohol Drinking
Alcohol induced disorders	Alcoholic intoxication	Law Enforcement
Mental Health	Mental Disorders	Mentally Ill Person
Depression	Mental Fatigue	Post Traumatic disorder
Diagnosis, Dual Psychiatry	Combat Disorders	

All articles and reports that meet inclusion criteria will have a manual search of their references to identify any additional articles. Peer reviewed articles identified through electronic automated searches that meet inclusion criteria will have their citations manually checked (title and abstract) for articles relevant to the review. Authors will be contacted where full text articles or reports are not available electronically or via the British Library.

### Study selection

PRISMA’s (Preferred Reporting Items for Systematic Reviews) ‘Four-Phase Flow Diagram’ [54] will be populated to provide a record of article source and article inclusion and exclusion during the four systematic review phases defined by PRISMA in their recent statement [54]. Details of all articles screened will be recorded using bibliography software.

### Quality assessment, grading of evidence and data extraction

Each stage will record standardized data, including details of design and methodology, participant characteristics and demographics, country, year of study, where published, and adverse events, comments or findings, if reported. Quality appraisal of the quantitative studies reviewed will depend on study type. Case–control or cohort studies will be evaluated using the corresponding Critical Appraisal Skills Programme (CASP) critical appraisal checklists [55]. Prevalence or incidence studies will be appraised using the criteria and methodological scoring system developed by Loney and colleagues [56]. The process and value in assessment of quality in qualitative research has long been debated and there are many tools for doing so [57]. This review will assess the quality of the primary research articles obtained using the CASP critical appraisal tool for qualitative studies. This tool will also be used to record the demographic data for the qualitative studies. A spread-sheet or simple database, one for each of the evaluation methodologies, will be created to document the quality assessments of the full text reviewed as well as the standardized information mentioned above.

Prior to data extraction all included articles will receive a coding which will classify the nature of the clinical presentation. Coding will define the principle clinical presentation as being either one of the mental health ICD-10 classifications, alcohol use, substance use, alcohol and substance use, or a mixed presentation. Additionally, if required, for articles examining alcohol and substance use the authors will further define a sub-classification process based on type of use. *A priori* classification will prevent any unintentional misclassifying of data after the analysis process has been concluded and interim results are identified.

Information obtained from data extraction will be tabulated. When available, statistical results will be identified from the quantitative research papers, while the themes, key concepts, narratives, and theories will be obtained from the qualitative reports using the process described by Thomas and Harden [58]. Where there is incomplete information an attempt will be made to contact the authors of papers to obtain the information.

The quality assessment and data extraction process will be conducted by a single researcher (Taylor), but will be cross-checked by a second reviewer (Parkes). Quality assessment and data extraction by a single researcher does introduce a potential for bias; however, the quality control cross-check process will reduce this. Disagreements, discrepancies or uncertainties over inclusion, quality assessment, or data extraction will be resolved by discussion or through the involvement of a third researcher from the team.

### Data synthesis and statistical analysis of systematic review

#### Analysis and synthesis 1

Stage 1 data will be tabulated and discussed in a narrative review. Analysis will include both direct and indirect comparisons. Assuming the test for homogeneity permits, meta-analysis of the ‘pooled’ quantitative data will measure the effect on relevant comparator outcomes, for example, the presence of mental health problems on criminal justice contact, the differences in clinical presentation (mental health variables compared with substance use variables), and sub-group differences. Analysis of pooled prevalence data and differences on incidence will also be conducted at this stage. SPSS software will be utilized for statistical calculations.

#### Analysis and synthesis 2

Stage 2 involves the ‘thematic synthesis’ of the aggregated qualitative data, as described by Thomas and Harden [58], which incorporates three stages: the ‘line by line’ coding of text, the development of descriptive themes and the generation of ‘analytical themes’. NVivo9 software will be used to support the coding and subsequent thematic analysis.

#### Analysis and synthesis 3

Stage 3 adopts a ‘mixed-method’ approach. From a qualitative orientation the thematic findings from Stage 2 will be juxtaposed with the sub-group and clinical presentation narrative results of Stage 1. A matrix approach will be used to conduct a comparative analysis between the two sets of findings examining these for matches, mismatches and gaps. An example of the general questions that will guide this section include whether there are specific veteran sub-groups, for example, combat exposure, that match the veteran views on what contributed to their contact with justice services. From a quantitative orientation, when comparing sub-groups, statistical analysis of effect sizes of presentations that match veteran views on what contributed to contact with criminal justice service services will be compared with those veterans whose views did not. For example, veterans with alcohol problems who state that drinking was a contributory factor to their criminal justice contact will be compared with veterans with drinking problems that did not so state or believe it was not contributory.

## Discussion

Combining diverse study types and answering different types of question in a systematic review can raise a number of methodological issues and, as such, require careful matching of study types to questions and methods of synthesis to the types of data obtained [52]. Given the broad range of questions posed within this review, it is likely that primary research papers and reports accessed would present a diverse spectrum of study types and methodological processes. For some of the questions the collation of data and analysis may be straightforward, for example, in identifying the prevalence of military veterans with either mental health or substance misuse problems in prison. Other questions may be more complicated and require a mixed method approach that can elicit a more detailed response. While the search strategy for this review is deliberately broad there is a possibility that articles and reports identified will be too heterogeneous, thereby limiting the opportunity to conduct direct statistical comparisons. Likewise, there is a concern that this is a subject area that is under-researched, with the possibility that too few formal quantitative and qualitative research studies or reports will be identified, thereby impacting on data analysis and synthesis of results and ultimately the ability to answer the primary and supplementary research questions.

Nonetheless, it is envisaged that this review will provide a greater understanding of the experiences and views of ex-military service personnel with mental health problems, including the problematic use of drugs and alcohol. It will also permit a greater understanding of whether their mental health problems contribute to their contact with criminal justice services; whether such contact differs from that of military veterans with good mental health; and, whether there are international differences. Additionally, given the recent withdrawal of service personnel from Iraq and the planned withdrawal of troops from Afghanistan, examining changes in criminal justice contact after previous key conflicts may provide an indicator as to whether, in the near future, a rise in military veteran contact with criminal justice services should be expected and planned for.

## Competing interests

The author JT will use the research and publication as part of his PhD studies. The remaining authors declare that they have no competing interests.

## Authors’ contributions

JT and TP contributed to the conception and design of the review and all authors were involved in the drafting of the protocol and have given their approval for publication. All authors read and approved the final manuscript.
